# The Antibacterial Activity of Human Amniotic Membrane against Multidrug-Resistant Bacteria Associated with Urinary Tract Infections: New Insights from Normal and Cancerous Urothelial Models

**DOI:** 10.3390/biomedicines9020218

**Published:** 2021-02-20

**Authors:** Taja Železnik Ramuta, Larisa Tratnjek, Aleksandar Janev, Katja Seme, Marjanca Starčič Erjavec, Mateja Erdani Kreft

**Affiliations:** 1 Institute of Cell Biology, Faculty of Medicine, University of Ljubljana, SI-1000 Ljubljana, Slovenia; taja.zeleznik@mf.uni-lj.si (T.Ž.R.); larisa.tratnjek@mf.uni-lj.si (L.T.); aleksandar.janev@mf.uni-lj.si (A.J.); 2 Institute of Microbiology and Immunology, Faculty of Medicine, University of Ljubljana, SI-1000 Ljubljana, Slovenia; katja.seme@mf.uni-lj.si; 3 Department of Biology, Biotechnical Faculty, University of Ljubljana, SI-1000 Ljubljana, Slovenia

**Keywords:** amniotic membrane homogenate, MRSA, multidrug-resistant bacteria, antimicrobial activity, antibiotic resistance, electron microscopy, urothelial cells, urinary bladder

## Abstract

Urinary tract infections (UTIs) represent a serious global health issue, especially due to emerging multidrug-resistant UTI-causing bacteria. Recently, we showed that the human amniotic membrane (hAM) could be a candidate for treatments and prevention of UPEC and *Staphylococcus aureus* infections. However, its role against multidrug-resistant bacteria, namely methicillin-resistant *S. aureus* (MRSA), extended-spectrum beta-lactamases (ESBL) producing *Escherichia coli* and *Klebsiella pneumoniae*, vancomycin-resistant *Enterococci* (VRE), carbapenem-resistant *Acinetobacter baumannii*, and *Pseudomonas aeruginosa* has not yet been thoroughly explored. Here, we demonstrate for the first time that the hAM homogenate had antibacterial activity against 7 out of 11 tested multidrug-resistant strains, the greatest effect was on MRSA. Using novel approaches, its activity against MRSA was further evaluated in a complex microenvironment of normal and cancerous urinary bladder urothelia. Even short-term incubation in hAM homogenate significantly decreased the number of bacteria in MRSA-infected urothelial models, while it did not affect the viability, number, and ultrastructure of urothelial cells. The hAM patches had no antibacterial activity against any of the tested strains, which further exposes the importance of the hAM preparation. Our study substantially contributes to basic knowledge on the antibacterial activity of hAM and reveals its potential to be used as an antibacterial agent against multidrug-resistant bacteria.

## 1. Introduction

The widespread use of antibiotics led to the development of bacterial resistance, which is associated with increased morbidity, mortality, and healthcare costs [1,2,3,4,5]. Nearly 15% of all prescribed antibiotics in the United States are used for the treatment of urinary tract infections (UTI), which are among the most common bacterial infections in humans and represent an important health problem [6,7], especially as high rates of antibiotic resistance among uropathogenic bacteria are found worldwide [3,5,8,9,10,11]. While years ago the antibiotic resistance was characteristic for nosocomial uropathogens and these were found mainly in patients with comorbidities (e.g., diabetes or reflux nephropathy), in recent years antibiotic resistance is commonly found also in community-acquired uropathogens [12].

Bacteria have evolved sophisticated mechanisms of drug resistance to ensure their survival and importantly, resistance to antibiotics can be achieved through multiple biochemical pathways [13]. Mechanisms of antibiotic resistance can be divided in the following categories: (a) Modifications of the antibiotic molecule (chemical alterations of the antibiotic [14,15], destruction of the antibiotic molecule [16,17]), (b) decreased antibiotic penetration and efflux (decreased permeability [18,19], efflux pumps [20,21]), (c) changes in target sites (target protection [22,23], modification of the target site [24,25,26], complete replacement or bypass of the target site [27,28,29,30,31,32]), (d) resistance due to global cell adaptations [13,33,34]. Infections with antibiotic-resistant uropathogens increase the risk for pyelonephritis, recurrent UTIs, renal disease, preterm birth, and also contribute to various complications in vulnerable patients undergoing other treatments [35,36]. Furthermore, an increasing number of bacterial strains are multidrug resistant, which further limits the impact of the available antimicrobial agents [33]. UTIs are one of the most common healthcare-associated infections and reports of multidrug-resistant bacteria causing UTIs [37], especially methicillin-resistant *Staphylococcus aureus* (MRSA) [38,39,40], ESBL-producing *Escherichia coli* [41,42,43] and *Klebsiella pneumoniae* [7,44,45], multidrug-resistant *Acinetobacter baumannii* [46,47,48], vancomycin-resistant *Enterococci* (VRE) [49,50,51], and *Pseudomonas aeruginosa* [46,52,53], are increasing. All of these bacteria were included on the list of priority pathogens for research and development of new antibiotics published by the World Health Organization (WHO) in 2017. Moreover, carbapenem-resistant *A. baumannii* and *P. aeruginosa*, carbapenem-resistant and ESBL-producing *Enterobacteriaceae* were listed as “Priority 1: Critical”. In addition, vancomycin-resistant *Enterococcus faecium*, methicillin-resistant, vancomycin-intermediate and vancomycin-resistant *Staphylococcus aureus* were listed as “Priority 2: High”.

The human amniotic membrane (hAM) is the innermost part of the placenta that provides the essential physiological environment for prenatal development [54,55,56]. The 0.02–0.5 mm thick membrane consists of a monolayer of amniotic epithelial cells, basement membrane, and stroma, which is further divided into the compact layer, the layer of amniotic mesenchymal stromal cells and the spongy layer [54,57]. The use of hAM in clinical practice is increasing, especially due to its promotion of epithelization [58,59,60] and decrease of scarring [61,62,63,64], immunomodulatory [65,66,67,68,69,70,71], anticarcinogenic [72,73,74,75,76,77,78,79,80,81], and antimicrobial activity [82,83,84,85,86,87]. Moreover, our previous studies demonstrated that hAM homogenates have potent antibacterial activity against selected uropathogenic bacteria, including uropathogenic *E. coli* (UPEC), *Staphylococcus saprophyticus,* and *S. aureus* [82,83], etc.

The objective of this study was to investigate the antibacterial activity of hAM patches and hAM homogenates against multidrug-resistant bacteria, namely MRSA, ESBL-producing *E. coli* and *K. pneumoniae*, vancomycin-resistant *Enterococci*, carbapenem-resistant *A. baumannii* and *P. aeruginosa*, which could be potential UTI-causing agents. Moreover, as the antibacterial effect of hAM homogenate on MRSA was so profound, its effect was further evaluated in a more complex microenvironment, specifically in MRSA-infected biomimetic in vitro models of normal and cancerous urinary bladder urothelium.

## 2. Materials and Methods

### 2.1. Microorganisms

Bacterial strains used in this study are listed in Table 1. The strains were grown in liquid Luria-Bertani (LB) broth (Formedium, Hunstanton, UK) overnight at 37 °C with aeration (100 rpm).

### 2.2. Biomimetic In Vitro Models of Normal and Cancerous Urinary Bladder Urothelium

The biomimetic in vitro model of normal urothelium was established using the normal porcine urothelial cells (NPU), as described previously [88,89,90]. Briefly, primary and secondary cultures of NPU cells were established from three porcine normal urinary bladders (biological replicates), which were obtained from a local abattoir, as described previously. Briefly, each bladder was cut into stripes and urothelial cells were gently scraped with a scalpel blade. Cells were seeded in tissue culture flasks and grown in the UroM medium, consisting of the MCDB153 (Sigma-Aldrich, St. Louis, MS, USA) and Advanced Dulbecco’s modified essential medium (Gibco, Thermo Fisher Scientific, Waltham, MA, USA; ratio 1:1), supplemented with 2.5% fetal bovine serum (Gibco, Thermo Fisher Scientific, Waltham, MA, USA), adenine (15 mg/mL; St. Louis, MS, USA), hydrocortisone (0.5 mg/mL; Sigma-Aldrich, St. Louis, MS, USA), phosphoethanolamine (0.1 M; Sigma-Aldrich, St. Louis, MS, USA), insulin (5 mg/mL; Sigma-Aldrich, St. Louis, MS, USA), and glutamax (4 mM; Gibco, Thermo Fisher Scientific, Waltham, MA, USA). Passages IV to XI of NPU cells were used for experiments at a seeding density of 1 × 10^5^ cells/cm^2^. For the establishment of the highly differentiated urothelial model, which resembles normoplastic urothelium in vivo, NPU cells were seeded on synthetic scaffolds (porous membrane with a pore diameter of 0.4 μm; BD Falcon, Corning, New York, NY, USA) at a seeding density of 1 × 10^5^ cells/cm^2^. After reaching confluence, the cells were cultured for an additional 3 weeks in the serum-free culture medium with an added physiological calcium concentration of 2.5 mM.

The biomimetic in vitro model of cancerous urothelium was established using cancer urothelial T24 cells, originating from the human invasive urothelial neoplasm (ATCC, USA), as described previously [90,91]. Briefly, T24 cells were seeded on synthetic scaffolds (porous membrane with a pore diameter of 0.4 μm; BD Falcon, Corning, New York, NY, USA) at a seeding density of 5 × 10^4^ cells/cm^2^. They were cultured in a culture medium, consisting of Advanced-Dulbecco’s modified essential medium (Gibco, Thermo Fisher Scientific, Waltham, MA, USA) and F12 medium (Sigma-Aldrich, St. Louis, MS, USA; ratio 1:1), supplemented with a 5% fetal bovine serum (Gibco, Thermo Fisher Scientific, Waltham, MA, USA) and glutamax (4 mM; Gibco, Thermo Fisher Scientific, Waltham, MA, USA). T24 cells were cultured for 1 week at 37 °C and 5% CO_2_. All of the cell cultures were maintained at 37 °C in a humidified atmosphere of 5% CO_2_.

### 2.3. The hAM Preparation

The preparation of hAM patches and hAM homogenates was carried out as described previously [82,83]. Briefly, hAM was first manually separated from the chorion and washed with a sterile phosphate-buffered saline (PBS). To prepare hAM patches, hAM was cut into pieces of approximately 1 × 1 cm and stored at 4 °C for a maximum of 6 h before use (fresh hAM; f-hAM patches) or cryopreserved in PBS at −80 °C (cryopreserved hAM; c-hAM patches). All of the cryopreserved samples went only through one freeze-thaw cycle.

To prepare the hAM homogenates, hAM was cut into pieces (3 × 3 cm), which were then mixed with sterile PBS (ratio one part of hAM pieces and three parts of sterile PBS) and homogenized in a homogenizer (Russell Hobbs, 21350-56, 400 W) for 3–4 min. For testing the antibacterial activity of hAM homogenate on in vitro urothelial models, the hAM homogenates were prepared in the NPU cells’ and T24 cells’ culture media rather than PBS. The culture medium consisting of the MCDB153 (Sigma-Aldrich, St. Louis, MS, USA) and Advanced Dulbecco’s modified essential medium (Gibco, Thermo Fisher Scientific, Waltham, MA, USA; ratio 1:1), supplemented with adenine (15 mg/mL; Sigma-Aldrich, St. Louis, MS, USA), hydrocortisone (0.5 mg/mL; Sigma-Aldrich, St. Louis, MS, USA), phosphoethanolamine (0.1 M; Sigma-Aldrich, St. Louis, MS, USA), insulin (5 mg/mL; Sigma-Aldrich, St. Louis, MS, USA), and glutamax (4 mM; Gibco, Thermo Fisher Scientific, Waltham, MA, USA) was used for the NPU cells and culture medium consisting of Advanced Dulbecco’s modified essential medium (Gibco, Thermo Fisher Scientific, Waltham, MA, USA; ratio 1:1), F12 medium (Sigma-Aldrich, St. Louis, MS, USA; ratio 1:1), supplemented with glutamax (4 mM; Gibco, Thermo Fisher Scientific, Waltham, MA, USA) was used for the T24 cells. Afterwards, the hAM homogenate was filtered through a nylon filter with a 1 mm pore diameter. The hAM homogenates were stored at 4 °C for a maximum of 6 h before use (f-hAM homogenate) or were cryopreserved at −80 °C (c-hAM homogenate). Before use, all the c-hAM homogenates were supplemented with CaCl_2_ (for NPU cells; the final concentration of CaCl_2_ was 2.5 mM) or 5% FBS (for T24 cells). All the cryopreserved samples went only through one freeze-thaw cycle.

### 2.4. Antibacterial Susceptibility Testing on Agar Plates Using the hAM Homogenate and Various Antibiotic Discs

Antibacterial susceptibility tests were performed using Muller-Hinton soft agar and Muller-Hinton agar plates (Formedium, Hunstanton, UK) with either hAM patches (f-hAM and c-hAM patches rinsed for 5 min in sterile PBS), hAM homogenate or antibiotic discs (trimethoprim 1.25 μg/sulfamethoxazole 23.75 μg, clindamycin 2 μg, erythromycin 15 μg, cefoxitin 30 μg, penicillin 1 U, and linezolid 10 μg). Bacterial strains (Table 1) were applied onto the plates in Muller-Hinton soft agar that was cooked at 100 °C for 10 min, cooled to 48 °C, and then inoculated with 100 μL of bacterial overnight culture. In the case of hAM patches, the patches were first placed on Muller-Hinton plates and subsequently the inoculated Muller-Hinton soft agar was poured over. In the case of hAM homogenate and antibiotic discs, the inoculated Muller-Hinton soft agar was first poured over the Muller-Hinton agar plates and then left to solidify for 5–10 min at room temperature. Afterwards, 5 and 10 μL of f-hAM and c-hAM homogenates (in triplicates for each volume) or antibiotic discs were placed on the agar plate. To determine the antibacterial activity in all three types of tests, all the plates were incubated at 35–37 °C for 24 h and afterwards investigated for the inhibition zones. The susceptibility of bacteria to the selected antibiotics was interpreted according to the EUCAST clinical breakpoints.

### 2.5. Analysis of the Antibacterial Activity of hAM Homogenate on Biomimetic In Vitro Models of Normal and Cancerous Urothelia Infected with MRSA

The in vitro urothelial models were incubated for 3 h at 35–37 °C in (a) the NPU cells’ culture medium (control) or the T24 cells’ culture medium (control), (b) the c-hAM homogenate, (c) the NPU cells’ culture medium or the T24 cells’ culture medium inoculated with MRSA (NCTC 12493; 20 μL of an overnight culture of MRSA was inoculated in 1 mL of a culture medium) and (d) the c-hAM homogenate inoculated with MRSA (NCTC 12493; 20 μL of an overnight culture of MRSA was inoculated in 1 mL of c-hAM homogenate).

After the incubation, the culture medium or hAM homogenate from biomimetic in vitro urothelial models infected with MRSA were collected and the number of live MRSA cells per ml was determined using the colony forming unit (CFU) quantification method. To quantify the number of bacteria either attached to the surface or endocytosed by the NPU and T24 cells, the biomimetic in vitro models were treated with the 0.5% Triton-X-100 solution in 0.9% NaCl for 20 min at room temperature and then the culture medium for the NPU or T24 cells was added, the cells were scraped from the surface, and the CFU quantification method was performed. All the dilutions for the CFU quantification method were prepared using the sterile physiological solution (0.9% NaCl) and 100 μL of each dilution was plated on the Muller-Hinton agar plates, and cultured for 24 h at 35–37 °C. Afterwards, the colonies were counted and the number of CFU/mL was established. All the experiments were performed in triplicates.

### 2.6. Cell Viability Assay

To evaluate the viability of NPU and T24 cells after the 3 h incubation, the culture medium or hAM homogenate were removed, and the cells were treated with TrypLE Select (Thermo Fisher Scientific, Waltham, MA, USA) and incubated at 37 °C and 5% CO_2_ until they detached from the surface. Afterwards, the cell suspension was centrifuged for 5 min at 200× *g*, then the pellet was resuspended in a culture medium, and the cells were mixed with the Trypan blue dye. The viability of NPU and T24 cells was obtained by counting the number of viable and Trypan blue-labelled dead cells.

### 2.7. Scanning and Transmission Electron Microscopy

All the biomimetic in vitro urothelial models were analyzed by scanning and transmission electron microscopy. The samples were prepared as described previously [88,92]. Briefly, samples for scanning electron microscopy were fixed with 2% formaldehyde and 2% glutaraldehyde in a 0.2 M cacodylate buffer (pH 7.4) for 3 h at 4 °C. Then, they were rinsed overnight in a 0.2 M cacodylate buffer at 4 °C and then post-fixed in 1% osmium tetroxide in a 0.2 M cacodylate buffer for 2 h at room temperature, followed by dehydration through a graded series of ethanol and then acetone. Thereafter, the specimens were immersed in HMDS (hexamethyldisilazane; Sigma-Aldrich, St. Louis, MS, USA), air-dried at room temperature, sputter-coated with gold, and examined at 30 kV with the Vega 3 scanning electron microscope (Tescan, Brno, Czech Republic).

For transmission electron microscopy (TEM), samples were fixed with 3% formaldehyde and 3% glutaraldehyde in a 0.1 M cacodylate buffer for 3 h at 4 °C. Afterwards, the samples were rinsed overnight in a 0.1 M cacodylate buffer at 4 °C and then post-fixed in 2% osmium tetroxide for 1 h at room temperature, followed by incubation in 2% uranyl acetate in H_2_O for 1 h at room temperature. Next, the samples were dehydrated in a graded series of ethanol and embedded in Epon (Serva Electrophoresis, Heidelberg, Germany). Furthermore, ultrathin sections were contrasted with uranyl acetate and lead citrate and examined with the CM100 transmission electron microscope (Philips, Eindhoven, Netherlands), operation voltage 80 kV, equipped with the CCD camera (AMT, Danvers, MA, USA).

### 2.8. Statistical Analysis

All the data shown herein are based on three to eight biological samples of hAM and six to 30 total technical repeats for each strain or biomimetic in vitro model for each assay. All information about the materials and methods used are available also in the Protocols.io database. All the data are presented as the mean ± standard error of mean (SEM). All the statistical analyses were performed using the GraphPad Prism 6 software (GraphPad Software, Inc., San Diego, CA, USA), when appropriate using the parametric one-way ANOVA with the post-hoc Tukey multiple comparisons test or the non-parametric Kruskal-Wallis test with post-hoc Dunn’s multiple comparisons test. *p*-values of < 0.05 were considered statistically significant.

## 3. Results

### 3.1. The hAM Patches Have No Antibacterial Activity against Selected Multidrug-Resistant Bacteria

**Figure 1:**
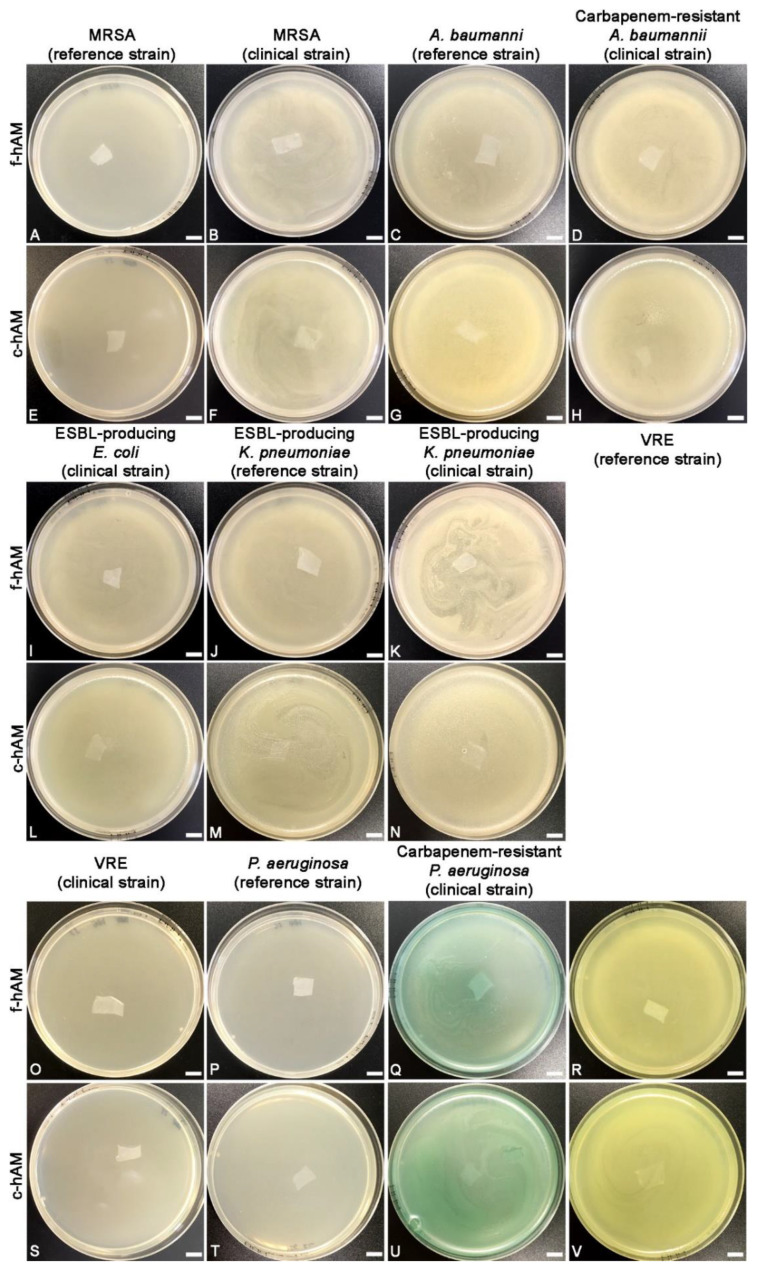
The human amniotic membrane (hAM) patches have no antibacterial activity against tested multidrug-resistant bacteria. Neither the application of fresh-hAM (f-hAM) patches (**A**–**D**, **I**–**K**, **O**–**R**) nor the application of cryopreserved hAM (c-hAM) patches (**E**–**H**,**L**–**N**,**S**–**V**) resulted in an inhibition zone in any of the tests. Data were obtained from three independent replications of experiments using three biological samples of hAM and three technical repeats for each biological sample and each independent replication of the experiment. Scale bars: 10 mm.

The f-hAM and c-hAM patches were embedded in Muller-Hinton soft agar, which was previously inoculated with bacterial strains listed in Table 1. After 24 h of incubation, all the plates were overgrown with bacteria (i.e., confluent growth), indicating that f-hAM and c-hAM patches have no antibacterial activity against the tested strains (Figure 1).

### 3.2. The hAM Homogenate Has Antibacterial Activity against Selected Multi-Drug Resistant Bacteria

**Figure 2:**
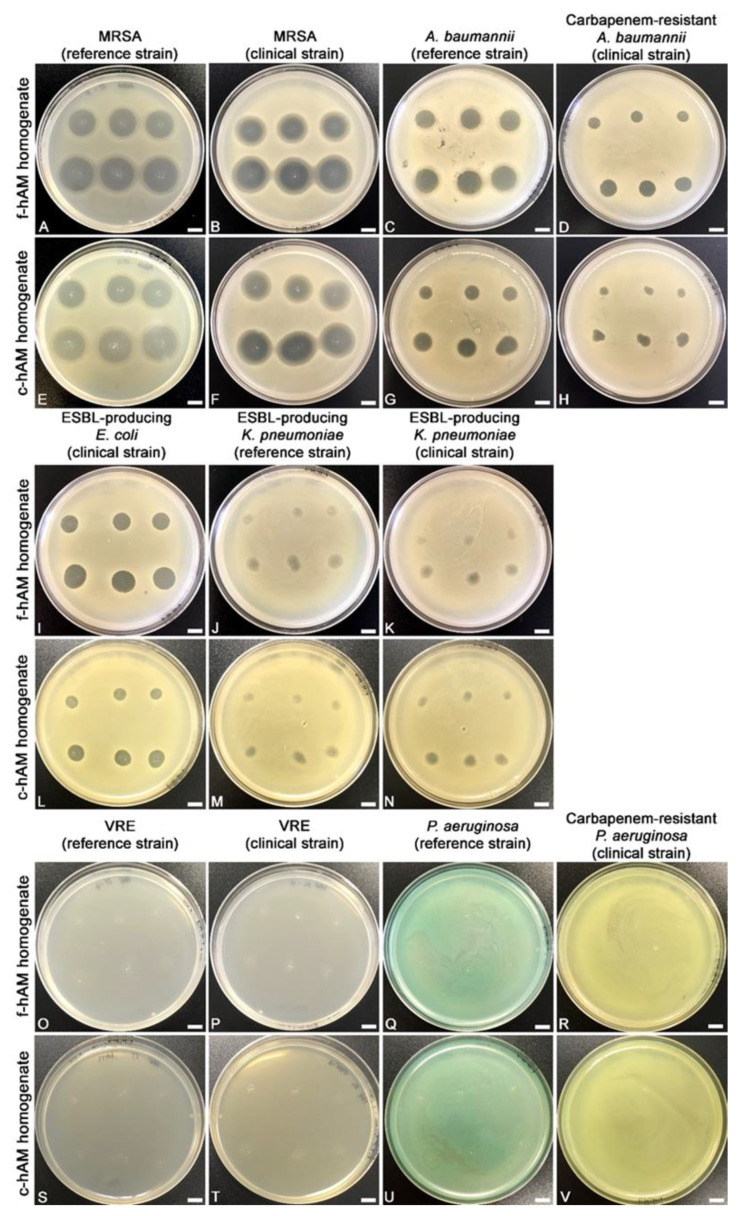
The f-hAM and c-hAM homogenates have antibacterial activity against seven out of 11 tested strains. (**A**,**B**,**E**,**F**,**I**,**L**) The application of f-hAM and c-hAM homogenates resulted in an inhibition zone in all the tests. (**C**,**D**,**G**,**H**) The application of f-hAM and c-hAM homogenates resulted in an inhibition zone in 75% of all the performed tests. (**J**,**K**,**M**,**N**) The application of f-hAM and c-hAM homogenates resulted in an inhibition zone in 25% of the performed tests. (**O**–**V**) The application of f-hAM and c-hAM homogenates did not result in an inhibition zone in any of the performed tests. Data were obtained from at least three independent replications of experiments using at least three biological samples of hAM and six technical repeats for each biological sample and each independent replication of the experiment. Scale bars: 10 mm.

**Figure 3:**
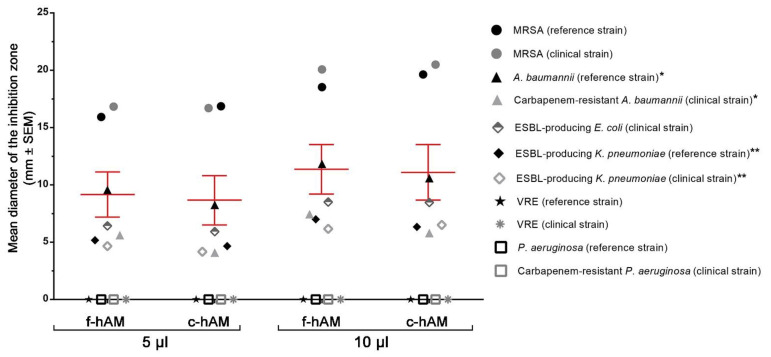
The range of antibacterial activity of f-hAM and c-hAM homogenates varies between the multidrug-resistant strains. The average antibacterial activity of f-hAM and c-hAM homogenates against all the susceptible strains is shown. The bars (red) show the mean diameter of the inhibition zone ± SEM (mm) of all the susceptible strains. On average, the application of f-hAM homogenate resulted in a larger inhibition zone than the application of c-hAM homogenate. Furthermore, a larger volume of hAM homogenates applied (10 µl) resulted in a larger inhibition zone than the smaller volume of hAM homogenates (5 µl). (*, **) Antibacterial activity of f-hAM and c-hAM homogenates was detected in 75% (*) or 25% (**) of all the performed tests and only these measurements were included in the mean diameter ± SEM (mm) of the inhibition zones.

The f-hAM and c-hAM homogenates had antibacterial activity against seven out of 11 tested strains. However, the potency of antibacterial activity varied between the tested strains. Namely, the hAM homogenate had potent antibacterial activity against three strains in all the performed tests (both tested strains of MRSA and the clinical ESBL-producing *E. coli* strain; Figure 2A,B,E,F,I,L and Figure 3, Table 2), while it had antibacterial activity against two strains in 75% of the performed tests (both tested strains of *A. baumannii*; Figure 2C,D,G,H and Figure 3, Table 2) and on two strains in 25% of the performed tests (both tested strains of ESBL-producing *K. pneumoniae*; Figure 2J,K,M,N, Table 2). In other words, in the case of both tested strains of *A. baumannii* the application of three out of four biological samples of hAM homogenates resulted in the inhibition zone, and for both strains of ESBL-producing *K. pneumoniae.* One out of four biological samples of hAM homogenates resulted in the inhibition zone. On the other hand, f-hAM and c-hAM homogenates did not have any antibacterial activity against reference and clinical strains of vancomycin-resistant *Enterococci* and on the reference and clinical strain of *P. aeruginosa*. Each result was obtained from at least three independent replications of experiments using three biological samples of hAM; each experiment was performed in six technical repeats for each strain.

The range of antibacterial activity of f-hAM and c-hAM homogenates varied between the tested strains. The average mean diameter of the inhibition zone for all susceptible strains was 9.2 ± 1.8 mm (5 μL) and 11.4 ± 2.0 mm (10 μL) when f-hAM was applied and 8.7 ± 2.0 mm (5 μL) and 11.1 ± 2.2 mm (10 μL) when c-hAM was applied (Figure 3, Table 2). Of all the tested strains, the reference and clinical strains of MRSA were most susceptible to f-hAM and c-hAM homogenates. Namely, the mean diameter of the inhibition zone for the reference strain of MRSA was 15.9 ± 0.5 mm (5 μL) and 18.5 ± 0.6 mm (10 μL; Figure 3, Table 2) when f-hAM was applied and was 16.9 ± 0.6 mm (5 μL) and 19.6 ± 0.6 mm (10 μL; Figure 3, Table 2) when c-hAM was applied. The clinical strain of MRSA was even more susceptible to f-hAM and c-hAM homogenates. The mean diameter of the inhibition zone was 16.83 ± 0.9 mm (5 μL) and 20.1 ± 0.9 mm (10 μL; Figure 3, Table 2) when f-hAM was applied and was 16.7 ± 0.9 mm (5 μL) and 20.5 ± 0.8 mm (10 μL; Figure 3, Table 2) when c-hAM was applied.

In all the susceptible bacterial strains, as little as 5 μL of f-hAM or c-hAM homogenates produced a pronounced inhibition zone. Moreover, the antibacterial activity was even more prominent when 10 μL of f-hAM or c-hAM homogenates were applied. Interestingly, the differences in the range of the inhibition zone when applying different volumes of hAM homogenates (5 or 10 μL) were not statistically significantly different (*p* > 0.05) for any of the susceptible strains with the exception of the c-hAM-treated clinical strain of MRSA, for which there was a statistically significant difference in the range of the inhibition zones when comparing the 5 and 10 μL of hAM homogenates applied (*p* < 0.05; Supplementary Table S1). Importantly, the differences in the range of the inhibition zone were also not statistically different when comparing the antibacterial activity of f-hAM or c-hAM (5 and 10 μL, respectively) (*p* < 0.05; Supplementary Table S1).

### 3.3. Comparison of the Antibacterial Activity of hAM Homogenates and Selected Antibiotics against MRSA

**Figure 4:**
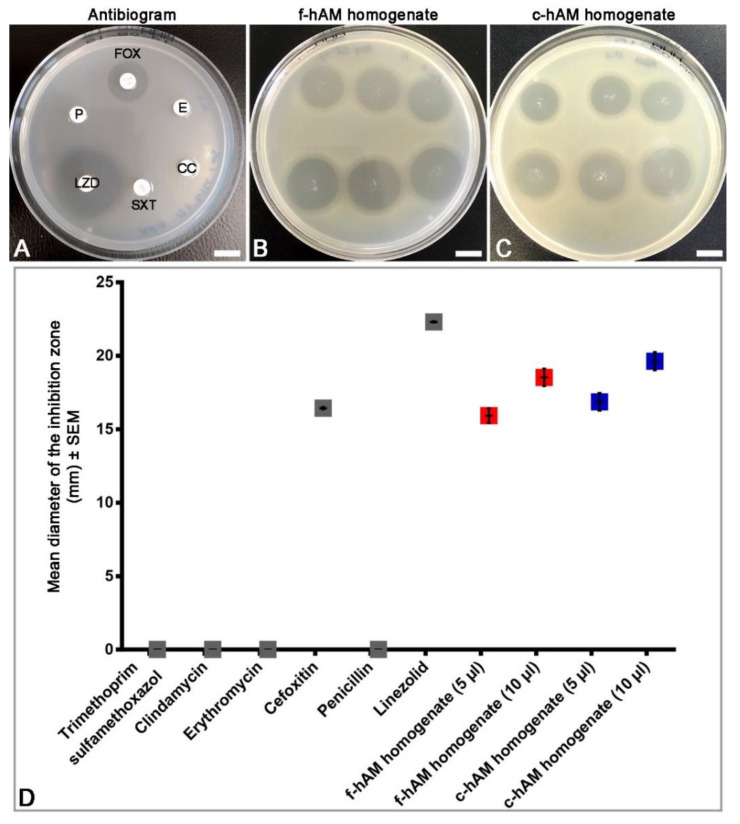
Comparison of the antibacterial activity of hAM homogenates and selected antibiotics against the reference strain of methicillin-resistant *S. aureus* (MRSA). (**A**,**D**) The reference strain of MRSA is resistant to trimethoprim/sulfamethoxazole, clindamycin, erythromycin, penicillin, and cefoxitin and is susceptible to linezolid. (**B**–**D**) The application of 5 and 10 μL f-hAM and c-hAM homogenates results in an inhibition zone. Scale bars: 10 mm.

The results of antibacterial susceptibility on solid agar demonstrated that the hAM homogenates had the most profound antibacterial activity against MRSA. Therefore, from this point on, the study focused on the antibacterial activity of hAM homogenates against the reference strain of MRSA, which was proved to be resistant to trimethoprim/sulfamethoxazole, clindamycin, erythromycin, and penicillin and susceptible to linezolid (according to the routine EUCAST and CLSI disc diffusion test, bacterial strains in which the inhibition zone around the 30 µg cefoxitin disc is equal to or larger than 22 mm, are considered methicillin susceptible) (Figure 4A,D). Using the antibacterial susceptibility assay, the antibacterial activity of f-hAM and c-hAM homogenates and selected antibiotics against this MRSA strain was evaluated. Even though the reference strain of MRSA is resistant to several antibiotics, the application of f-hAM and c-hAM homogenates resulted in a pronounced inhibition zone. Furthermore, the application of 5 and 10 µL of f-hAM homogenates resulted in the inhibition zones with the mean diameter of 15.9 ± 0.5 mm and 18.53 ± 0.6 mm, respectively. Similarly, the application of 5 and 10 µL of c-hAM homogenates resulted in the inhibition zones with the mean diameter of 16.8 ± 0.6 mm and 19.6 ± 0.6 mm, respectively (Figure 4B–D). Data were obtained from three biological samples of hAM and one independent replication of the experiment using antibiotic discs; each experiment was performed in 3–6 technical repeats.

### 3.4. The c-hAM Homogenate Decreases the Number of Bacteria in Biomimetic In Vitro Models of the Normal and Cancerous Urothelium

**Figure 5:**
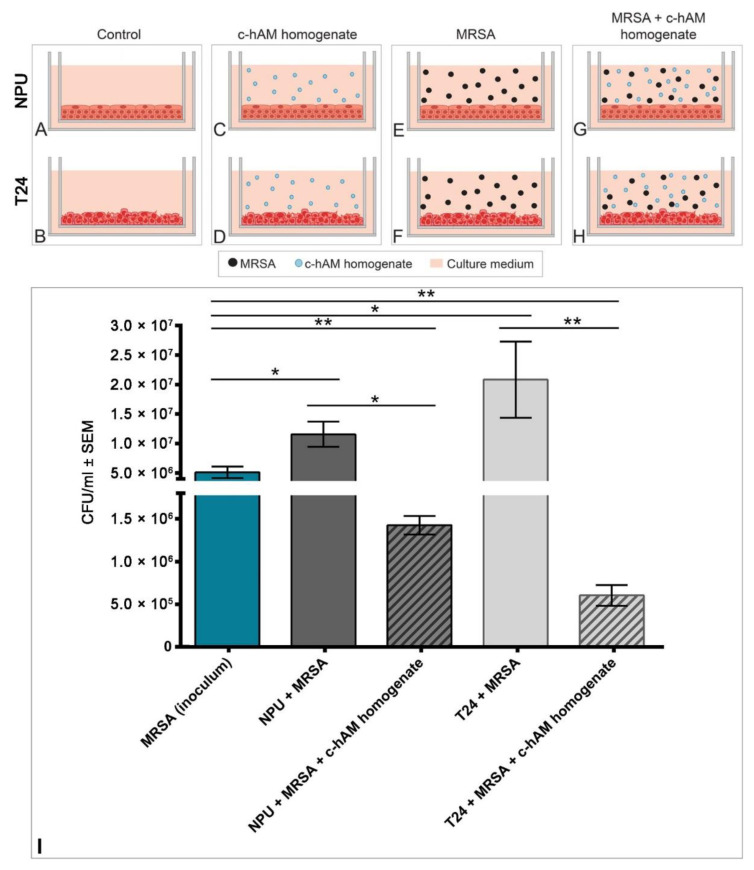
The effect of c-hAM homogenate on MRSA-infected biomimetic in vitro models of the normal and cancerous urothelium. (**A**–**H**) Scheme of the experiment. The normal porcine urothelial (NPU) and T24 cells were incubated for 3 h in the (**A**,**B**) culture medium (control), (**C**,**D**) c-hAM homogenate, (**E**,**F**) culture medium inoculated with MRSA, (**G**,**H**) c-hAM homogenate inoculated with MRSA. (**I**) The number of bacteria in MRSA-infected samples, incubated in the presence or absence of c-hAM homogenate. The c-hAM homogenate significantly decreased the number of bacteria in biomimetic in vitro models of the normal and cancerous urothelium. * *p* < 0.05; ** *p* < 0.001.

Since f-hAM and c-hAM homogenates had the largest effect on MRSA, this study focused on further evaluation of the antibacterial activity of hAM on the reference strain MRSA in a more complex microenvironment. Moreover, since there was no statistically significant difference in the range of the inhibition zone caused by the f-hAM or c-hAM homogenates (Supplementary Table S1), the c-hAM homogenate was used for further experiments as it is more relevant for potential clinical use. Biomimetic in vitro models of the normal urothelium (NPU cells) and cancerous urothelium (T24 cells) were prepared and inoculated with the reference strain of MRSA (6.0 × 10^6^ ± 9.5 × 10^5^ CFU/mL) for 3 h in the presence or absence of c-hAM homogenate. The following groups of samples were analyzed: (1) NPU (Figure 5A) and T24 cells (Figure 5B) incubated in the culture medium (control), (2) NPU (Figure 5C) and T24 cells (Figure 5D) incubated in the c-hAM homogenate, (3) NPU (Figure 5E) and T24 cells (Figure 5F) incubated in the culture medium inoculated with MRSA, and (4) NPU (Figure 5G) and T24 cells (Figure 5H) incubated in the c-hAM homogenate inoculated with MRSA (Figure 5G,H).

Using the CFU quantification method, it was shown that the treatment with the c-hAM homogenate significantly decreased the number of bacteria in the biomimetic in vitro models of the normal and cancerous urothelium (Figure 5I; *p* < 0.05). After the 3-h incubation, 93% fewer bacteria were detected in the normal urothelium, treated with c-hAM, than in the untreated normal urothelium. Similarly, 98% fewer bacteria were detected in the c-hAM-treated cancerous urothelium than in the untreated one. Moreover, after the treatment, there were fewer viable bacteria in the NPU and T24 cells than in the inoculum, indicating the bactericidal mechanism of action of the c-hAM homogenate (Figure 5I). Data were obtained from four independent replications of experiments using four biological samples of hAM; each experiment was performed in three technical repeats for each condition.

### 3.5. A Short-Term Incubation in c-hAM Homogenate Does Not Affect the Cell Viability or Ultrastructure of Biomimetic In Vitro Models of the Normal and Cancerous Urothelium

**Figure 6:**
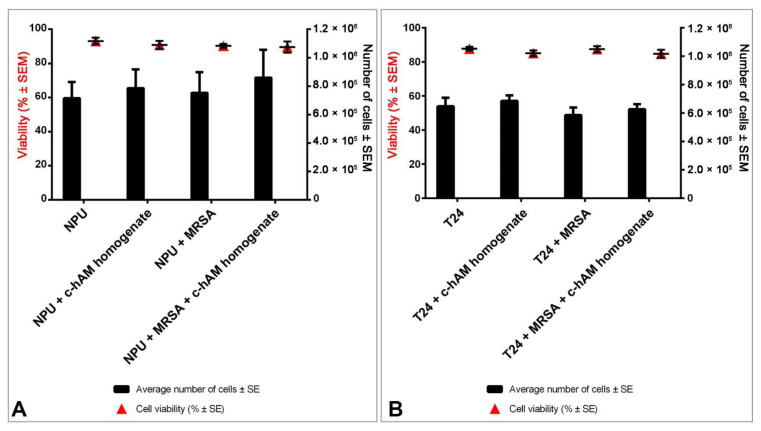
The effect of short-term incubation in the c-hAM homogenate and/or MRSA on the viability of biomimetic in vitro models of the normal and cancerous urothelium. The NPU (**A**) and T24 cells (**B**) maintained a high cell viability in all conditions and there were no statistically significant differences (*p* > 0.05) in cell viability between the treated and non-treated NPU and T24 cells. There were also no statistically significant differences in the number of viable cells between the treated and non-treated NPU and T24 cells. Data presented here show the percentage of viable cells ± SEM and the mean number of cells ± SEM for each sample. Data were obtained from three independent replications of experiments using three biological samples of hAM; each experiment was performed in two technical repeats for each condition.

After the 3-h incubation, the viability of cells comprising the biomimetic in vitro models of normal and cancerous urothelium was evaluated. The viability of NPU cells in all four groups varied between 89.6 ± 3.3% (NPU cells incubated in the c-hAM homogenate inoculated with MRSA) and 93.2 ± 1.9% (NPU cells incubated in the culture medium), but differences were not statistically significant (*p* > 0.05; Figure 6A). Moreover, the analysis of the number of viable NPU cells per each model showed that the differences between the samples were not statistically significant (*p* > 0.05; Figure 6A). Similarly, the viability of T24 cells in all four groups varied only between 84.7 ± 2.3% (T24 cells incubated in the c-hAM homogenate inoculated with MRSA) and 87.8 ± 1.1% (T24 cells incubated in the culture medium), and the differences were not statistically significant (*p* > 0.05; Figure 6B). Moreover, the differences in the number of viable T24 cells per each model were also not statistically significant (Figure 6B).

**Figure 7:**
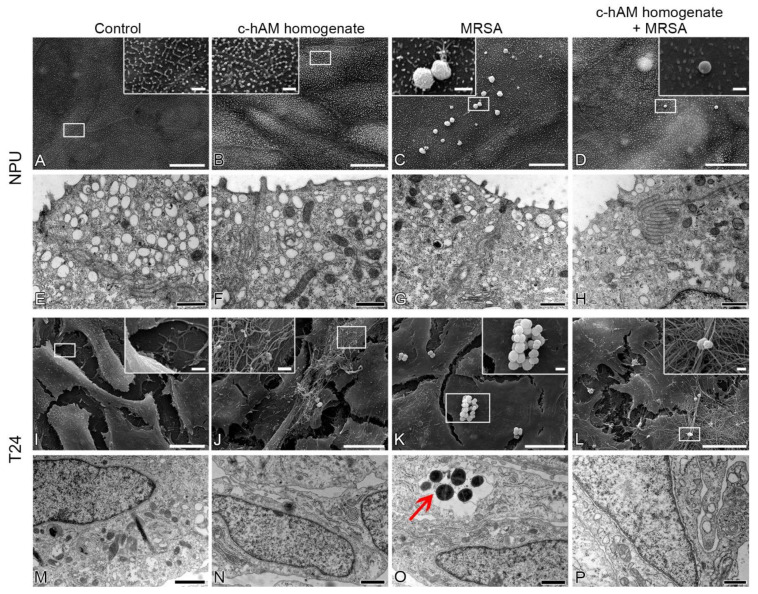
The effect of short-term incubation in the c-hAM homogenate and/or MRSA on the ultrastructure of biomimetic in vitro models of the normal and cancerous urothelium. (**A**,**B**,**E**,**F**) Short-term (3 h) treatment of the NPU cells with the c-hAM homogenate does not affect the ultrastructure of NPU cells. (**C**,**D**,**G,H**) During the 3 h incubation in the culture medium inoculated with MRSA, individual bacteria or small aggregates of MRSA attached to the surface of NPU cells. During the 3 h incubation in the c-hAM homogenate inoculated with MRSA, a smaller number of individual bacteria attached to the surface of NPU cells. (**I**,**J**,**M**,**N**) A short-term treatment of the T24 cells with the c-hAM homogenate does not affect the ultrastructure of T24 cells. (**K**,**O**) During the 3 h incubation in the culture medium inoculated with MRSA, aggregates of MRSA attached to the surface of the T24 cells and some of them were endocytosed by the T24 cells. (**L**,**P**) During the 3 h incubation in the c-hAM homogenate inoculated with MRSA, a smaller number of individual bacteria or small aggregates of bacteria attached to the surface of T24 cells. Large insets framed with white lines (**A**–**D**,**I**–**L**) show the enlarged areas of the corresponding small white-framed insets. Data were obtained from three independent replications of experiments using three biological samples of hAM. Arrow: An aggregate of MRSA in the endosomal compartment. Scale bars: (**A**–**D**,**I**–**L**) 10 µm; (enlarged areas in **A**–**D**,**I**–**L**) 1 µm; (**E**–**H**,**M**–**P**) 600 nm.

Using the scanning and transmission electron microscopy, the effect of a short-term (3 h) incubation of the NPU and T24 cells with the c-hAM homogenate and/or MRSA was evaluated (Figure 7). After the 3 h incubation of MRSA in the culture medium, the bacteria attached to the surface of NPU cells most commonly in the form of individual cells or small aggregates, but no bacteria endocytosed by the NPU cells were detected (Figure 7C,D,G,H). In the case of T24 cells, after the 3 h incubation of MRSA in the culture medium, the bacteria formed aggregates on the surface of T24 cells (Figure 7K,L) and some of the bacteria were endocytosed by the T24 cells (Figure 7O). Next, after the 3 h incubation of MRSA in the c-hAM homogenate, a lower number of bacteria attached to the surface and/or endocytosed by the NPU and T24 cells was observed (Figure 7D,H,L,P) in comparison to the number of attached and/or endocytosed bacteria in samples where MRSA was incubated in the culture medium, which is in accordance with the results shown in Figure 5. Moreover, MRSA attached to the surface of NPU cells individually and to the surface of T24 cells mainly in small aggregates (Figure 7D,L).

Furthermore, the effect of the 3 h incubation in the c-hAM homogenate on the ultrastructure of NPU and T24 cells was also evaluated (Figure 7). The short-term incubation in the c-hAM homogenate did not affect the ultrastructure of NPU and T24 cells. Interestingly, the c-hAM homogenate did not attach to the surface of NPU cells (Figure 7B,D), while it attached to the surface of T24 cells, limiting the contact of MRSA with T24 cells (Figure 7J,L).

## 4. Discussion

Due to the emergence and spread of bacteria, resistant to antibiotics, the treatment of UTIs is becoming increasingly difficult. Alarmingly, the number of UTIs, caused by multidrug-resistant bacteria is also rising, especially in the hospital setting [7,93,94,95]. Therefore, there is a great need for the development of novel antimicrobial agents.

The antibacterial activity of hAM was first reported by Talmi et al. in 1991 [96]. This finding was supported by several other studies, which demonstrated the antibacterial activity of hAM patches [86,87,96,97,98,99], the hAM extract [84,87,100], and the hAM-derived conditioned medium [85,101]. Studies have shown that human amniotic membrane epithelial cells and human amniotic membrane mesenchymal stromal cells secrete antimicrobial molecules, which are a part of the innate immune system. The α and β defensins possess antibacterial, antiviral, and antifungal activity and the Whey acidic peptide (WAP) motif containing proteins, which include the secretory leukocyte protease inhibitor (SLPI) and elafin, have been shown to have antimicrobial properties and also anti-protease activity [102,103,104,105,106,107].

Our research group showed that the hAM homogenates have potent antibacterial activity against several uropathogenic bacteria, including some clinical strains of multidrug resistant uropathogenic *E. coli* [82,83]. However, this is the first study in which the antibacterial activity of hAM against a plethora of multidrug-resistant bacteria was analyzed and to the best of our knowledge, the antibacterial activity of hAM has never been investigated before in a complex microenvironment.

### 4.1. The hAM Patches Have No Antibacterial Activity against Multidrug-Resistant Bacteria

First, we tested whether f-hAM and c-hAM patches have an antibacterial activity against the tested strains. No antibacterial activity under or around the hAM patches was detected, which is in accordance with our previous study showing that hAM patches do not have an antibacterial activity against selected strains of Gram-positive and Gram-negative uropathogenic bacteria [82]. Interestingly, there are several reports of antibacterial activity of hAM patches in the literature [86,87,96,98,99,108] and we attribute this disparity to differences in hAM handling and sample preparation. We hypothesize that during the removal of amnion from chorion and subsequent handling, some damage to the hAM-derived cells might occur, which could result in the release of antimicrobial molecules. Hence, if the hAM-derived cells remain intact during the preparation of hAM, the antimicrobial molecules are not released and no antibacterial activity of hAM patches is detected. These discrepancies indicate a great need for the standardization of protocols for the preparation of hAM to ensure the best quality of hAM-derived preparations.

### 4.2. The hAM Homogenates Have Antibacterial Activity against Several Multidrug-Resistant Bacteria

The hAM homogenates had robust antibacterial activity against three out of 11 tested strains (reference and clinical strains of MRSA and clinical strain of ESBL-producing *E. coli*), with an application of all the biological samples of hAM homogenates resulting in inhibition zones in all the tests. This is an important finding as MRSA and ESBL-producing *E. coli* are among the most common pathogens causing healthcare-associated infections and are exceedingly difficult to treat due to their multidrug resistance [109]. Consequently, these patients often require a more invasive treatment approach, namely intravenous rather than oral application of antibiotics [110,111].

Next, our results also show that there is no statistically significant difference in the range of the inhibition zone when comparing the application of the same volume of f-hAM and c-hAM homogenates. This is certainly very important when considering the implementation of hAM homogenates in clinical practice, since it is much easier to ensure the sufficient supply of cryopreserved than fresh hAM-derived preparations. Furthermore, future studies must ascertain the proper concentrations of hAM homogenate for clinical application to ensure the best clinical outcome and also evaluate infectious agents as “susceptible”, “intermediate” or “resistant” to hAM homogenate according to the ISO standard 20,776 for determination of the resistance of infectious agents.

Interestingly, in the case of reference and clinical strains of *A. baumannii* and ESBL-producing *K. pneumoniae*, not all hAM biological samples demonstrated antibacterial activity. Moreover, none of the tested biological samples of hAM had any antimicrobial effect on the reference and clinical strains of VRE and *P. aeruginosa*. These findings demonstrate how crucial donor heterogeneity is and point to the immense need for further research to elucidate which molecules contribute to the antimicrobial activity and their mechanism of action. Since it is currently not known which molecules, in addition to the innate immune system molecules mentioned above, are crucial for the antimicrobial activity of hAM, it is impossible to improve donor selection as it is not clear which factors induce the synthesis and secretion of the hAM antimicrobial molecules. Therefore, to ensure robust results, additional studies are needed to determine the chemical composition of hAM-derived preparations and how the age of the donor, health status, and gestational age at delivery affect the antimicrobial activity of hAM. Moreover, special attention must also be focused on the standardization of hAM preparation and storage protocols, which crucially affect the range of antimicrobial activity of hAM homogenates.

### 4.3. The hAM Homogenate Demonstrated Antibacterial Activity against MRSA-Infected Biomimetic Models of the Normal and Cancerous Urothelium

UTIs represent more than 30% of all healthcare-associated infections [112]. Most of the UTIs are caused by the uropathogenic *E. coli*, although MRSA is becoming an increasingly important uropathogen in the hospital setting [38,39,113]. It is disconcerting that 22% of patients in which urine MRSA was detected, developed invasive infections with MRSA in the next 12 months, such as endocarditis, osteomyelitis, and skin and soft tissue infections [114]. Therefore, symptomatic as well as asymptomatic patients who test positive for MRSA in the urine need to be treated, and this is of the utmost importance especially for patients who will undergo surgical treatment [38,115]. Since the treatment of infections caused by MRSA is limited by their multidrug resistance, this highlights the need for the development of novel antimicrobial agents that are effective against multidrug-resistant bacteria.

Using antibacterial susceptibility tests, we have shown that hAM homogenates are highly effective against MRSA. To test their efficacy in a more complex microenvironment, we applied c-hAM homogenates to biomimetic in vitro models of normal and cancerous urothelium in the presence or absence of MRSA. Not only was the number of bacteria in c-hAM homogenate-treated urothelia statistically significantly lower than in the untreated urothelia, but it was even lower than the number of bacteria in the inoculum, indicating the bactericidal activity of c-hAM homogenate. This is in agreement with our previous study which showed that the 2- to 4-fold diluted c-hAM homogenate had a bactericidal effect on *S. aureus* [83].

A short-term (3 h) incubation of the normal and cancerous urothelium with MRSA does not affect the viability of normal and cancer urothelial cells. These results show that a short-term application of the hAM homogenate would decrease the concentration of uropathogenic bacteria, while having no toxic effect on the epithelial cells. Particularly, the toxicity is a shortcoming of some antibiotics, e.g., fluoroquinolones, which induce the cell cycle arrest in the S phase or the S/G_2_ transition, which leads the eukaryotic cells into apoptosis [116]. These results are the first step towards evaluating the safety of hAM homogenates, which will be crucial for the translation of hAM-derived preparations from bench to bedside.

Our previous studies showed that hAM scaffolds enable the development of urothelium with molecular and ultrastructural properties comparable to those of native urothelium [92] and that hAM scaffolds enriched with the urinary bladder fibroblasts promote the re-epithelization of the urothelial injury [117]. Therefore, we believe that the hAM homogenate has the potential not only to decrease the number of bacteria, but also to promote the regeneration of the urothelium, allowing for more rapid tissue regeneration after bacterial infection.

Furthermore, our research group also demonstrated that the cells and extracellular matrix of hAM hinder the growth and invasive potential of bladder urothelial cancer cells [81]. Here, we evaluated the effect of hAM homogenate only after a short-term (3 h) incubation, while in the previous study we demonstrated that the most prominent anticancer effect of hAM scaffolds is reached in the first three days of treatment. Thus, we hypothesize that the hAM homogenate may also have the anticancer effect, and our next study will evaluate the effect of long-term treatments and multiple repetitions of short-term treatments with hAM homogenate on cancer cells. Since bacterial infections are one of the most common complications in immunosuppressed cancer patients and are often associated with considerable morbidity, mortality, and financial burden [118], it would be very beneficial to develop a therapeutic agent that would have both antimicrobial and anticancer effects.

The use of hAM has several benefits, as its use is ethically acceptable, it has many unique properties that promote tissue regeneration, it is easy to prepare, and is also a low-cost solution. On the other hand, the use of hAM has some challenges and limitations, such as the limited shelf life of hAM-derived preparations and the heterogeneity between biological samples of hAM. To sum up, the hAM homogenates have great potential to be used as antibacterial agents, and further research is needed to elucidate which hAM-derived molecules cause the potent antimicrobial effect. In addition, this knowledge will promote faster and safer translation of hAM homogenates into clinical practice.

## Figures and Tables

**Table 1:** List of bacterial strains used in the experiments.

Strains	Relevant Genotype and/or Phenotype Features	Gram Stain	Reference/Source
*Staphylococcus aureus*	Reference strain; methicillin-resistant *mecA*-positive	Gram-positive	NCTC 12493
*Staphylococcus aureus*	Clinical strain; methicillin-resistant	Gram-positive	Blood culture
*Acinetobacter baumannii*	Reference strain	Gram-negative	ATCC 33604
*Acinetobacter baumannii*	Clinical strain; carbapenem-resistant	Gram-negative	Endotracheal aspirate
*Escherichia coli*	Clinical strain; extended-spectrum beta-lactamase positive	Gram-negative	Blood culture
*Klebsiella pneumoniae*	Reference strain; SHV-18 extended-spectrum beta-lactamase-producer	Gram-negative	ATCC 700603
*Klebsiella pneumoniae*	Clinical strain; extended-spectrum beta-lactamase positive	Gram-negative	Blood culture
*Enterococcus faecalis*	Reference strain; vancomycin-resistant, *vanB*-positive strain	Gram-positive	ATCC 51299
*Enterococcus faecalis*	Clinical strain; vancomycin-resistant	Gram-positive	Urine
*Pseudomonas aeruginosa*	Reference strain	Gram-negative	ATCC 27853
*Pseudomonas aeruginosa*	Clinical strain; carbapenem-resistant	Gram-negative	Endotracheal aspirate

**Table 2:** The range of antibacterial activity of f-hAM and c-hAM homogenates varies between multidrug-resistant strains.

Bacterial Strain	f-hAM Homogenate		c-hAM Homogenate	
	5 μL	10 μL	5 μL	10 μL
	*mean diameter of the inhibition zone ± SEM (mm)*			
MRSA (reference strain)	15.9 ± 0.5	18.5 ± 0.6	16.9 ± 0.6	19.6 ± 0.6
MRSA (clinical strain)	16.8 ± 0.9	20.1 ± 0.9	16.7 ± 0.9	20.5 ± 0.8
ESBL-producing *E. coli* (clinical strain)	6.4 ± 0.5	8.5 ± 0.6	5.9 ± 0.6	8.5 ± 0.6
*A. baumannii* (reference strain) *	9.6 ± 0.4	11.8 ± 0.4	8.3 ± 0.5	10.6 ± 0.5
Carbapenem-resistant *A. baumannii* (clinical strain) *	5.6 ± 0.3	7.4 ± 0.4	4.1 ± 0.6	5.8 ± 0.9
ESBL-producing *K. pneumoniae* (reference strain) **	5.2 ± 0.3	7.0 ± 0.4	4.7 ± 0.2	6.3 ± 0.2
ESBL-producing *K. pneumoniae* (clinical strain) **	4.7 ± 0.2	6.2 ± 0.3	4.2 ± 0.3	6.5 ± 0.2
VRE (reference strain)	–			
VRE (clinical strain)	–			
*P. aeruginosa* (reference strain)	–			
*P. aeruginosa* (clinical strain)	–			

Shown are the mean diameters ± SEM (mm) of the inhibition zones due to the antibacterial activity of f-hAM and c-hAM homogenates against the tested strains. (–) No inhibition zone. (*, **) Antibacterial activity of f-hAM and c-hAM homogenates was detected in 75% (*) or 25% (**) of all the performed tests and only these measurements were included in the mean diameter ± SEM (mm) of the inhibition zones.

## Data Availability

The data presented in this study are available on request from the corresponding author. All information regarding the materials and methods used are also available in the Protocols.io database [119].

## Supplementary Materials

The following are available online at https://www.mdpi.com/2227-9059/9/2/218/s1. Table S1: The analysis of variance (ANOVA) of the antimicrobial effect of f-hAM and c-hAM homogenates for all the susceptible strains.
