# Supplementary material for: The Antibacterial Activity of Human Amniotic Membrane against Multidrug-Resistant Bacteria Associated with Urinary Tract Infections: New Insights from Normal and Cancerous Urothelial Models
Source: Biomedicines. 2021 Feb 20;9(2):218. doi: 10.3390/biomedicines9020218 (PMC7924402; doi:10.3390/biomedicines9020218)
Supplement: Supplementary file 1 [file biomedicines-09-00218-s001.pdf]

Supplementary Material

The Antibacterial Activity of Human Amniotic Membrane against Multidrug-Resistant Bacteria Associated with Urinary Tract Infections: New Insights from Normal and Cancerous Urothelial Models

Taja Železnik Ramuta[1], Larisa Tratnjek[1], Aleksandar Janev[1], Katja Seme[2], Marjanca Starčič Erjavec[3]*, Mateja Erdani Kreft[1]*

[1] Institute of Cell Biology, Faculty of Medicine, University of Ljubljana, Ljubljana, Slovenia

[2] Institute of Immunology and Microbiology, Faculty of Medicine, University of Ljubljana, Ljubljana, Slovenia

[3] Department of Biology, Biotechnical Faculty, University of Ljubljana, Ljubljana, Slovenia

*Correspondence: marjanca.starcic.erjavec@bf.uni-lj.si (M.S.E.); mateja.erdani@mf.uni-lj.si (M.E.K.)

Supplementary Table 1. The analysis of variance (ANOVA) of the antimicrobial effect of f-hAM and c-hAM homogenates for all susceptible strains. The parametric one-way ANOVA with post-hoc Tukey's multiple comparisons test was used when there were more than 20 measurements per condition and the data followed normal distribution (all analyses for reference and clinical strains of MRSA). The non-parametric Kruskal-Wallis test with post-hoc Dunn's multiple comparisons test was used when there were 6-20 measurements per condition and the data followed normal distribution(all analyses for reference and clinical strains of *A. baumannii*, ESBL-producing *E. coli* and ESBL-producing *K. pneumoniae*). $p<0.05$ values were considered statistically significant.

**Analysis of variance (ANOVA)**	
**Tukey's multiple comparisons test**	**Adjusted P Value**
MRSA (reference strain) + 5 µl of f-hAM homogenate vs. MRSA (reference strain) + 10 µl of f-hAM homogenate	0.2299
MRSA (reference strain) + 5 µl of f-hAM homogenate vs. MRSA (clinical strain) + 5 µl of f-hAM homogenate	0.9904
MRSA (reference strain) + 5 µl of f-hAM homogenate vs. MRSA (clinical strain) + 10 µl of f-hAM homogenate	0.0035
MRSA (reference strain) + 5 µl of f-hAM homogenate vs. MRSA (reference strain) + 5 µl of c-hAM homogenate	0.9881
MRSA (reference strain) + 5 µl of f-hAM homogenate vs. MRSA (reference strain) + 10 µl of c-hAM homogenate	0.0144
MRSA (reference strain) + 5 µl of f-hAM homogenate vs. MRSA (clinical strain) + 5 µl of c-hAM homogenate	0.9964
MRSA (reference strain) + 5 µl of f-hAM homogenate vs. MRSA (clinical strain) + 10 µl of c-hAM homogenate	0.0007
MRSA (reference strain) + 10 µl of f-hAM homogenate vs. MRSA (clinical strain) + 5 µl of f-hAM homogenate	0.7549
MRSA (reference strain) + 10 µl of f-hAM homogenate vs. MRSA (clinical strain) + 10 µl of f-hAM homogenate	0.8398
MRSA (reference strain) + 10 µl of f-hAM homogenate vs. MRSA (reference strain) + 5 µl of c-hAM homogenate	0.7732
MRSA (reference strain) + 10 µl of f-hAM homogenate vs. MRSA (reference strain) + 10 µl of c-hAM homogenate	0.9695
MRSA (reference strain) + 10 µl of f-hAM homogenate vs. MRSA (clinical strain) + 5 µl of c-hAM homogenate	0.6765
MRSA (reference strain) + 10 µl of f-hAM homogenate vs. MRSA (clinical strain) + 10 µl of c-hAM homogenate	0.5925
MRSA (clinical strain) + 5 µl of f-hAM homogenate vs. MRSA (clinical strain) + 10 µl of f-hAM homogenate	0.0546
MRSA (clinical strain) + 5 µl of f-hAM homogenate vs. MRSA (reference strain) + 5 µl of c-hAM homogenate	> 0.9999
MRSA (clinical strain) + 5 µl of f-hAM homogenate vs. MRSA (reference strain) + 10 µl of c-hAM homogenate	0.1535
MRSA (clinical strain) + 5 µl of f-hAM homogenate vs. MRSA (clinical strain) + 5 µl of c-hAM homogenate	> 0.9999
MRSA (clinical strain) + 5 µl of f-hAM homogenate vs. MRSA (clinical strain) + 10 µl of c-hAM homogenate	0.016
MRSA (clinical strain) + 10 µl of f-hAM homogenate vs. MRSA (reference strain) + 5 µl of c-hAM homogenate	0.0596
MRSA (clinical strain) + 10 µl of f-hAM homogenate vs. MRSA (reference strain) + 10 µl of c-hAM homogenate	> 0.9999
MRSA (clinical strain) + 10 µl of f-hAM homogenate vs. MRSA (clinical strain) + 5 µl of c-hAM homogenate	0.0382

MRSA (clinical strain) + 10 µl of f-hAM homogenate vs. MRSA (clinical strain) + 10 µl of c-hAM homogenate	> 0.9999
MRSA (reference strain) + 5 µl of c-hAM homogenate vs. MRSA (reference strain) + 10 µl of c-hAM homogenate	0.1647
MRSA (reference strain) + 5 µl of c-hAM homogenate vs. MRSA (clinical strain) + 5 µl of c-hAM homogenate	> 0.9999
MRSA (reference strain) + 5 µl of c-hAM homogenate vs. MRSA (clinical strain) + 10 µl of c-hAM homogenate	0.0177
MRSA (reference strain) + 10 µl of c-hAM homogenate vs. MRSA (clinical strain) + 5 µl of c-hAM homogenate	0.1142
MRSA (reference strain) + 10 µl of c-hAM homogenate vs. MRSA (clinical strain) + 10 µl of c-hAM homogenate	0.9924
MRSA (clinical strain) + 5 µl of c-hAM homogenate vs. MRSA (clinical strain) + 10 µl of c-hAM homogenate	0.0106
**Dunn's multiple comparisons test**	**Adjusted P Value**
A. *baumannii* (reference strain) + 5 µl of f-hAM homogenate vs. A. *baumannii* (reference strain) + 10 µl of f-hAM homogenate	> 0.9999
A. *baumannii* (reference strain) + 5 µl of f-hAM homogenate vs. A. *baumannii* (clinical strain) + 5 µl of f-hAM homogenate	0.0009
A. *baumannii* (reference strain) + 5 µl of f-hAM homogenate vs. *A. baumannii* (clinical strain) + 10 µl of f-hAM homogenate	> 0.9999
A. *baumannii* (reference strain) + 5 µl of f-hAM homogenate vs. ESBL-producing *E. coli* (clinical strain) + 5 µl of f-hAM homogenate	0.0346
A. *baumannii* (reference strain) + 5 µl of f-hAM homogenate vs. ESBL-producing *E. coli* (clinical strain) + 10 µl of f-hAM homogenate	> 0.9999
A. *baumannii* (reference strain) + 5 µl of f-hAM homogenate vs. ESBL-producing *K. pneumoniae* (reference strain) + 5 µl of f-hAM homogenate	0.0372
A. *baumannii* (reference strain) + 5 µl of f-hAM homogenate vs. ESBL-producing *K. pneumoniae* (reference strain) + 10 µl of f-hAM homogenate	> 0.9999
A. *baumannii* (reference strain) + 5 µl of f-hAM homogenate vs. ESBL-producing *K. pneumoniae* (clinical strain) + 5 µl of f-hAM homogenate	0.0043
A. *baumannii* (reference strain) + 5 µl of f-hAM homogenate vs. ESBL-producing *K. pneumoniae* (clinical strain) + 10 µl of f-hAM homogenate	> 0.9999
A. *baumannii* (reference strain) + 5 µl of f-hAM homogenate vs. A. *baumannii* (reference strain) + 5 µl of c-hAM homogenate	> 0.9999
A. *baumannii* (reference strain) + 5 µl of f-hAM homogenate vs. A. *baumannii*(reference strain) + 10 µl of c-hAM homogenate	> 0.9999
A. *baumannii* (reference strain) + 5 µl of f-hAM homogenate vs. A. *baumannii*(clinical strain) + 5 µl of c-hAM homogenate	0.0002
A. *baumannii* (reference strain) + 5 µl of f-hAM homogenate vs. *A. baumannii* (clinical strain) + 10 µl of c-hAM homogenate	> 0.9999
A. *baumannii* (reference strain) + 5 µl of f-hAM homogenate vs. ESBL-producing *E. coli* (clinical strain) + 5 µl of c-hAM homogenate	0.01
A. *baumannii* (reference strain) + 5 µl of f-hAM homogenate vs. ESBL-producing *E. coli* (clinical strain) + 10 µl of c-hAM homogenate	> 0.9999
A. *baumannii* (reference strain) + 5 µl of f-hAM homogenate vs. ESBL-producing*K. pneumoniae*(reference strain) + 5 µl of c-hAM homogenate	0.0043
A. *baumannii* (reference strain) + 5 µl of f-hAM homogenate vs. ESBL-producing *K. pneumoniae*(reference strain) + 10 µl of c-hAM homogenate	> 0.9999
A. *baumannii* (reference strain) + 5 µl of f-hAM homogenate vs. ESBL-producing *K. pneumoniae*(clinical strain) + 5 µl of c-hAM homogenate	0.0008
A. *baumannii* (reference strain) + 5 µl of f-hAM homogenate vs. ESBL-producing *K. pneumoniae* (clinical strain) + 10 µl of c-hAM homogenate	> 0.9999
A. *baumannii* (reference strain) + 10 µl of f-hAM homogenate vs. A. *baumannii* (clinical strain) + 5 µl of f-hAM homogenate	< 0.0001

A. *baumannii* (reference strain) + 10 µl of f-hAM homogenate vs. *A. baumannii* (clinical strain) + 10 µl of f-hAM homogenate	0.0154
A. *baumannii* (reference strain) + 10 µl of f-hAM homogenate vs. ESBL-producing *E. coli* (clinical strain) + 5 µl of f-hAM homogenate	< 0.0001
A. *baumannii* (reference strain) + 10 µl of f-hAM homogenate vs. ESBL-producing *E. coli* (clinical strain) + 10 µl of f-hAM homogenate	0.1905
A. *baumannii* (reference strain) + 10 µl of f-hAM homogenate vs. ESBL-producing *K. pneumoniae* (reference strain) + 5 µl of f-hAM homogenate	0.0002
A. *baumannii* (reference strain) + 10 µl of f-hAM homogenate vs. ESBL-producing *K. pneumoniae* (reference strain) + 10 µl of f-hAM homogenate	0.4182
A. *baumannii* (reference strain) + 10 µl of f-hAM homogenate vs. ESBL-producing *K. pneumoniae* (clinical strain) + 5 µl of f-hAM homogenate	< 0.0001
A. *baumannii* (reference strain) + 10 µl of f-hAM homogenate vs. ESBL-producing *K. pneumoniae* (clinical strain) + 10 µl of f-hAM homogenate	0.0216
A. *baumannii* (reference strain) + 10 µl of f-hAM homogenate vs. A. *baumannii* (reference strain) + 5 µl of c-hAM homogenate	0.1496
A. *baumannii* (reference strain) + 10 µl of f-hAM homogenate vs. A. *baumannii*(reference strain) + 10 µl of c-hAM homogenate	> 0.9999
A. *baumannii* (reference strain) + 10 µl of f-hAM homogenate vs. A. *baumannii*(clinical strain) + 5 µl of c-hAM homogenate	< 0.0001
A. *baumannii* (reference strain) + 10 µl of f-hAM homogenate vs. *A. baumannii* (clinical strain) + 10 µl of c-hAM homogenate	0.0654
A. *baumannii* (reference strain) + 10 µl of f-hAM homogenate vs. ESBL-producing *E. coli* (clinical strain) + 5 µl of c-hAM homogenate	< 0.0001
A. *baumannii* (reference strain) + 10 µl of f-hAM homogenate vs. ESBL-producing *E. coli* (clinical strain) + 10 µl of c-hAM homogenate	0.1663
A. *baumannii* (reference strain) + 10 µl of f-hAM homogenate vs. ESBL-producing*K. pneumoniae*(reference strain) + 5 µl of c-hAM homogenate	< 0.0001
A. *baumannii* (reference strain) + 10 µl of f-hAM homogenate vs. ESBL-producing *K. pneumoniae*(reference strain) + 10 µl of c-hAM homogenate	0.0446
A. *baumannii* (reference strain) + 10 µl of f-hAM homogenate vs. ESBL-producing *K. pneumoniae*(clinical strain) + 5 µl of c-hAM homogenate	< 0.0001
A. *baumannii* (reference strain) + 10 µl of f-hAM homogenate vs. ESBL-producing *K. pneumoniae* (clinical strain) + 10 µl of c-hAM homogenate	0.0843
A. *baumannii* (clinical strain) + 5 µl of f-hAM homogenate vs. *A. baumannii* (clinical strain) + 10 µl of f-hAM homogenate	> 0.9999
A. *baumannii* (clinical strain) + 5 µl of f-hAM homogenate vs. ESBL-producing *E. coli* (clinical strain) + 5 µl of f-hAM homogenate	> 0.9999
A. *baumannii* (clinical strain) + 5 µl of f-hAM homogenate vs. ESBL-producing *E. coli* (clinical strain) + 10 µl of f-hAM homogenate	0.1503
A. *baumannii* (clinical strain) + 5 µl of f-hAM homogenate vs. ESBL-producing *K. pneumoniae* (reference strain) + 5 µl of f-hAM homogenate	> 0.9999
A. *baumannii* (clinical strain) + 5 µl of f-hAM homogenate vs. ESBL-producing *K. pneumoniae* (reference strain) + 10 µl of f-hAM homogenate	> 0.9999
A. *baumannii* (clinical strain) + 5 µl of f-hAM homogenate vs. ESBL-producing *K. pneumoniae* (clinical strain) + 5 µl of f-hAM homogenate	> 0.9999
A. *baumannii* (clinical strain) + 5 µl of f-hAM homogenate vs. ESBL-producing *K. pneumoniae* (clinical strain) + 10 µl of f-hAM homogenate	> 0.9999
A. *baumannii* (clinical strain) + 5 µl of f-hAM homogenate vs. A. *baumannii* (reference strain) + 5 µl of c-hAM homogenate	0.1913
A. *baumannii* (clinical strain) + 5 µl of f-hAM homogenate vs. A. *baumannii*(reference strain) + 10 µl of c-hAM homogenate	< 0.0001
A. *baumannii* (clinical strain) + 5 µl of f-hAM homogenate vs. A. *baumannii*(clinical strain) + 5 µl of c-hAM homogenate	> 0.9999
A. *baumannii* (clinical strain) + 5 µl of f-hAM homogenate vs. *A. baumannii* (clinical strain) + 10 µl of c-hAM homogenate	> 0.9999

A. *baumannii* (clinical strain) + 5 µl of f-hAM homogenate vs. ESBL-producing *E. coli* (clinical strain) + 5 µl of c-hAM homogenate	> 0.9999
A. *baumannii* (clinical strain) + 5 µl of f-hAM homogenate vs. ESBL-producing *E. coli* (clinical strain) + 10 µl of c-hAM homogenate	0.1723
A. *baumannii* (clinical strain) + 5 µl of f-hAM homogenate vs. ESBL-producing*K. pneumoniae*(reference strain) + 5 µl of c-hAM homogenate	> 0.9999
A. *baumannii* (clinical strain) + 5 µl of f-hAM homogenate vs. ESBL-producing *K. pneumoniae*(reference strain) + 10 µl of c-hAM homogenate	> 0.9999
A. *baumannii* (clinical strain) + 5 µl of f-hAM homogenate vs. ESBL-producing *K. pneumoniae*(clinical strain) + 5 µl of c-hAM homogenate	> 0.9999
A. *baumannii* (clinical strain) + 5 µl of f-hAM homogenate vs. ESBL-producing *K. pneumoniae* (clinical strain) + 10 µl of c-hAM homogenate	> 0.9999
A. baumannii (clinical strain) + 10 µl of f-hAM homogenate vs. ESBL-producing *E. coli* (clinical strain) + 5 µl of f-hAM homogenate	> 0.9999
A. baumannii (clinical strain) + 10 µl of f-hAM homogenate vs. ESBL-producing *E. coli* (clinical strain) + 10 µl of f-hAM homogenate	> 0.9999
A. baumannii (clinical strain) + 10 µl of f-hAM homogenate vs. ESBL-producing *K. pneumoniae* (reference strain) + 5 µl of f-hAM homogenate	> 0.9999
A. baumannii (clinical strain) + 10 µl of f-hAM homogenate vs. ESBL-producing *K. pneumoniae* (reference strain) + 10 µl of f-hAM homogenate	> 0.9999
A. baumannii (clinical strain) + 10 µl of f-hAM homogenate vs. ESBL-producing *K. pneumoniae* (clinical strain) + 5 µl of f-hAM homogenate	> 0.9999
A. baumannii (clinical strain) + 10 µl of f-hAM homogenate vs. ESBL-producing *K. pneumoniae* (clinical strain) + 10 µl of f-hAM homogenate	> 0.9999
A. baumannii (clinical strain) + 10 µl of f-hAM homogenate vs. A. *baumannii* (reference strain) + 5 µl of c-hAM homogenate	> 0.9999
A. baumannii (clinical strain) + 10 µl of f-hAM homogenate vs. A. *baumannii*(reference strain) + 10 µl of c-hAM homogenate	0.4734
A. baumannii (clinical strain) + 10 µl of f-hAM homogenate vs. A. *baumannii*(clinical strain) + 5 µl of c-hAM homogenate	> 0.9999
A. baumannii (clinical strain) + 10 µl of f-hAM homogenate vs. *A. baumannii* (clinical strain) + 10 µl of c-hAM homogenate	> 0.9999
A. baumannii (clinical strain) + 10 µl of f-hAM homogenate vs. ESBL-producing *E. coli* (clinical strain) + 5 µl of c-hAM homogenate	> 0.9999
A. baumannii (clinical strain) + 10 µl of f-hAM homogenate vs. ESBL-producing *E. coli* (clinical strain) + 10 µl of c-hAM homogenate	> 0.9999
A. baumannii (clinical strain) + 10 µl of f-hAM homogenate vs. ESBL-producing*K. pneumoniae*(reference strain) + 5 µl of c-hAM homogenate	> 0.9999
A. baumannii (clinical strain) + 10 µl of f-hAM homogenate vs. ESBL-producing *K. pneumoniae*(reference strain) + 10 µl of c-hAM homogenate	> 0.9999
A. baumannii (clinical strain) + 10 µl of f-hAM homogenate vs. ESBL-producing *K. pneumoniae*(clinical strain) + 5 µl of c-hAM homogenate	0.5569
A. baumannii (clinical strain) + 10 µl of f-hAM homogenate vs. ESBL-producing *K. pneumoniae* (clinical strain) + 10 µl of c-hAM homogenate	> 0.9999
ESBL-producing *E. coli* (clinical strain) + 5 µl of f-hAM homogenate vs. ESBL-producing *E. coli* (clinical strain) + 10 µl of f-hAM homogenate	> 0.9999
ESBL-producing *E. coli* (clinical strain) + 5 µl of f-hAM homogenate vs. ESBL-producing *K. pneumoniae* (reference strain) + 5 µl of f-hAM homogenate	> 0.9999
ESBL-producing *E. coli* (clinical strain) + 5 µl of f-hAM homogenate vs. ESBL-producing *K. pneumoniae* (reference strain) + 10 µl of f-hAM homogenate	> 0.9999
ESBL-producing *E. coli* (clinical strain) + 5 µl of f-hAM homogenate vs. ESBL-producing *K. pneumoniae* (clinical strain) + 5 µl of f-hAM homogenate	> 0.9999
ESBL-producing *E. coli* (clinical strain) + 5 µl of f-hAM homogenate vs. ESBL-producing *K. pneumoniae* (clinical strain) + 10 µl of f-hAM homogenate	> 0.9999
ESBL-producing *E. coli* (clinical strain) + 5 µl of f-hAM homogenate vs. A. *baumannii* (reference strain) + 5 µl of c-hAM homogenate	> 0.9999

ESBL-producing *E. coli* (clinical strain) + 5 µl of f-hAM homogenate vs. A. *baumannii*(reference strain) + 10 µl of c-hAM homogenate	0.0006
ESBL-producing *E. coli* (clinical strain) + 5 µl of f-hAM homogenate vs. A. *baumannii*(clinical strain) + 5 µl of c-hAM homogenate	> 0.9999
ESBL-producing *E. coli* (clinical strain) + 5 µl of f-hAM homogenate vs. *A. baumannii* (clinical strain) + 10 µl of c-hAM homogenate	> 0.9999
ESBL-producing *E. coli* (clinical strain) + 5 µl of f-hAM homogenate vs. ESBL-producing *E. coli* (clinical strain) + 5 µl of c-hAM homogenate	> 0.9999
ESBL-producing *E. coli* (clinical strain) + 5 µl of f-hAM homogenate vs. ESBL-producing *E. coli* (clinical strain) + 10 µl of c-hAM homogenate	> 0.9999
ESBL-producing *E. coli* (clinical strain) + 5 µl of f-hAM homogenate vs. ESBL-producing*K. pneumoniae*(reference strain) + 5 µl of c-hAM homogenate	> 0.9999
ESBL-producing *E. coli* (clinical strain) + 5 µl of f-hAM homogenate vs. ESBL-producing *K. pneumoniae*(reference strain) + 10 µl of c-hAM homogenate	> 0.9999
ESBL-producing *E. coli* (clinical strain) + 5 µl of f-hAM homogenate vs. ESBL-producing *K. pneumoniae*(clinical strain) + 5 µl of c-hAM homogenate	> 0.9999
ESBL-producing *E. coli* (clinical strain) + 5 µl of f-hAM homogenate vs. ESBL-producing *K. pneumoniae* (clinical strain) + 10 µl of c-hAM homogenate	> 0.9999
ESBL-producing *E. coli* (clinical strain) + 10 µl of f-hAM homogenate vs. ESBL-producing *K. pneumoniae* (reference strain) + 5 µl of f-hAM homogenate	0.9682
ESBL-producing *E. coli* (clinical strain) + 10 µl of f-hAM homogenate vs. ESBL-producing *K. pneumoniae* (reference strain) + 10 µl of f-hAM homogenate	> 0.9999
ESBL-producing *E. coli* (clinical strain) + 10 µl of f-hAM homogenate vs. ESBL-producing *K. pneumoniae* (clinical strain) + 5 µl of f-hAM homogenate	0.1636
ESBL-producing *E. coli* (clinical strain) + 10 µl of f-hAM homogenate vs. ESBL-producing *K. pneumoniae* (clinical strain) + 10 µl of f-hAM homogenate	> 0.9999
ESBL-producing *E. coli* (clinical strain) + 10 µl of f-hAM homogenate vs. A. *baumannii* (reference strain) + 5 µl of c-hAM homogenate	> 0.9999
ESBL-producing *E. coli* (clinical strain) + 10 µl of f-hAM homogenate vs. A. *baumannii*(reference strain) + 10 µl of c-hAM homogenate	> 0.9999
ESBL-producing *E. coli* (clinical strain) + 10 µl of f-hAM homogenate vs. A. *baumannii*(clinical strain) + 5 µl of c-hAM homogenate	0.0443
ESBL-producing *E. coli* (clinical strain) + 10 µl of f-hAM homogenate vs. *A. baumannii* (clinical strain) + 10 µl of c-hAM homogenate	> 0.9999
ESBL-producing *E. coli* (clinical strain) + 10 µl of f-hAM homogenate vs. ESBL-producing *E. coli* (clinical strain) + 5 µl of c-hAM homogenate	> 0.9999
ESBL-producing *E. coli* (clinical strain) + 10 µl of f-hAM homogenate vs. ESBL-producing *E. coli* (clinical strain) + 10 µl of c-hAM homogenate	> 0.9999
ESBL-producing *E. coli* (clinical strain) + 10 µl of f-hAM homogenate vs. ESBL-producing*K. pneumoniae*(reference strain) + 5 µl of c-hAM homogenate	0.1636
ESBL-producing *E. coli* (clinical strain) + 10 µl of f-hAM homogenate vs. ESBL-producing *K. pneumoniae*(reference strain) + 10 µl of c-hAM homogenate	> 0.9999
ESBL-producing *E. coli* (clinical strain) + 10 µl of f-hAM homogenate vs. ESBL-producing *K. pneumoniae*(clinical strain) + 5 µl of c-hAM homogenate	0.0405
ESBL-producing *E. coli* (clinical strain) + 10 µl of f-hAM homogenate vs. ESBL-producing *K. pneumoniae* (clinical strain) + 10 µl of c-hAM homogenate	> 0.9999
ESBL-producing *K. pneumoniae* (reference strain) + 5 µl of f-hAM homogenate vs. ESBL-producing *K. pneumoniae* (reference strain) + 10 µl of f-hAM homogenate	> 0.9999
ESBL-producing *K. pneumoniae* (reference strain) + 5 µl of f-hAM homogenate vs. ESBL-producing *K. pneumoniae* (clinical strain) + 5 µl of f-hAM homogenate	> 0.9999
ESBL-producing *K. pneumoniae* (reference strain) + 5 µl of f-hAM homogenate vs. ESBL-producing *K. pneumoniae* (clinical strain) + 10 µl of f-hAM homogenate	> 0.9999
ESBL-producing *K. pneumoniae* (reference strain) + 5 µl of f-hAM homogenate vs. A. *baumannii* (reference strain) + 5 µl of c-hAM homogenate	> 0.9999
ESBL-producing *K. pneumoniae* (reference strain) + 5 µl of f-hAM homogenate vs. A. *baumannii*(reference strain) + 10 µl of c-hAM homogenate	0.0044

ESBL-producing *K. pneumoniae* (reference strain) + 5 µl of f-hAM homogenate vs. A. *baumannii*(clinical strain) + 5 µl of c-hAM homogenate	> 0.9999
ESBL-producing *K. pneumoniae* (reference strain) + 5 µl of f-hAM homogenate vs. *A. baumannii* (clinical strain) + 10 µl of c-hAM homogenate	> 0.9999
ESBL-producing *K. pneumoniae* (reference strain) + 5 µl of f-hAM homogenate vs. ESBL-producing *E. coli* (clinical strain) + 5 µl of c-hAM homogenate	> 0.9999
ESBL-producing *K. pneumoniae* (reference strain) + 5 µl of f-hAM homogenate vs. ESBL-producing *E. coli* (clinical strain) + 10 µl of c-hAM homogenate	> 0.9999
ESBL-producing *K. pneumoniae* (reference strain) + 5 µl of f-hAM homogenate vs. ESBL-producing*K. pneumoniae*(reference strain) + 5 µl of c-hAM homogenate	> 0.9999
ESBL-producing *K. pneumoniae* (reference strain) + 5 µl of f-hAM homogenate vs. ESBL-producing *K. pneumoniae*(reference strain) + 10 µl of c-hAM homogenate	> 0.9999
ESBL-producing *K. pneumoniae* (reference strain) + 5 µl of f-hAM homogenate vs. ESBL-producing *K. pneumoniae*(clinical strain) + 5 µl of c-hAM homogenate	> 0.9999
ESBL-producing *K. pneumoniae* (reference strain) + 5 µl of f-hAM homogenate vs. ESBL-producing *K. pneumoniae* (clinical strain) + 10 µl of c-hAM homogenate	> 0.9999
ESBL-producing *K. pneumoniae* (reference strain) + 10 µl of f-hAM homogenate vs. ESBL-producing *K. pneumoniae* (clinical strain) + 5 µl of f-hAM homogenate	> 0.9999
ESBL-producing *K. pneumoniae* (reference strain) + 10 µl of f-hAM homogenate vs. ESBL-producing *K. pneumoniae* (clinical strain) + 10 µl of f-hAM homogenate	> 0.9999
ESBL-producing *K. pneumoniae* (reference strain) + 10 µl of f-hAM homogenate vs. A. *baumannii* (reference strain) + 5 µl of c-hAM homogenate	> 0.9999
ESBL-producing *K. pneumoniae* (reference strain) + 10 µl of f-hAM homogenate vs. A. *baumannii*(reference strain) + 10 µl of c-hAM homogenate	> 0.9999
ESBL-producing *K. pneumoniae* (reference strain) + 10 µl of f-hAM homogenate vs. A. *baumannii*(clinical strain) + 5 µl of c-hAM homogenate	> 0.9999
ESBL-producing *K. pneumoniae* (reference strain) + 10 µl of f-hAM homogenate vs. *A. baumannii* (clinical strain) + 10 µl of c-hAM homogenate	> 0.9999
ESBL-producing *K. pneumoniae* (reference strain) + 10 µl of f-hAM homogenate vs. ESBL-producing *E. coli* (clinical strain) + 5 µl of c-hAM homogenate	> 0.9999
ESBL-producing *K. pneumoniae* (reference strain) + 10 µl of f-hAM homogenate vs. ESBL-producing *E. coli* (clinical strain) + 10 µl of c-hAM homogenate	> 0.9999
ESBL-producing *K. pneumoniae* (reference strain) + 10 µl of f-hAM homogenate vs. ESBL-producing*K. pneumoniae*(reference strain) + 5 µl of c-hAM homogenate	> 0.9999
ESBL-producing *K. pneumoniae* (reference strain) + 10 µl of f-hAM homogenate vs. ESBL-producing *K. pneumoniae*(reference strain) + 10 µl of c-hAM homogenate	> 0.9999
ESBL-producing *K. pneumoniae* (reference strain) + 10 µl of f-hAM homogenate vs. ESBL-producing *K. pneumoniae*(clinical strain) + 5 µl of c-hAM homogenate	> 0.9999
ESBL-producing *K. pneumoniae* (reference strain) + 10 µl of f-hAM homogenate vs. ESBL-producing *K. pneumoniae* (clinical strain) + 10 µl of c-hAM homogenate	> 0.9999
ESBL-producing *K. pneumoniae* (clinical strain) + 5 µl of f-hAM homogenate vs. ESBL-producing *K. pneumoniae* (clinical strain) + 10 µl of f-hAM homogenate	> 0.9999
ESBL-producing *K. pneumoniae* (clinical strain) + 5 µl of f-hAM homogenate vs. A. *baumannii* (reference strain) + 5 µl of c-hAM homogenate	0.1929
ESBL-producing *K. pneumoniae* (clinical strain) + 5 µl of f-hAM homogenate vs. A. *baumannii*(reference strain) + 10 µl of c-hAM homogenate	0.0004
ESBL-producing *K. pneumoniae* (clinical strain) + 5 µl of f-hAM homogenate vs. A. *baumannii*(clinical strain) + 5 µl of c-hAM homogenate	> 0.9999
ESBL-producing *K. pneumoniae* (clinical strain) + 5 µl of f-hAM homogenate vs. *A. baumannii* (clinical strain) + 10 µl of c-hAM homogenate	0.7758
ESBL-producing *K. pneumoniae* (clinical strain) + 5 µl of f-hAM homogenate vs. ESBL-producing *E. coli* (clinical strain) + 5 µl of c-hAM homogenate	> 0.9999
ESBL-producing *K. pneumoniae* (clinical strain) + 5 µl of f-hAM homogenate vs. ESBL-producing *E. coli* (clinical strain) + 10 µl of c-hAM homogenate	0.1795
ESBL-producing *K. pneumoniae* (clinical strain) + 5 µl of f-hAM homogenate vs. ESBL-producing*K. pneumoniae*(reference strain) + 5 µl of c-hAM homogenate	> 0.9999

ESBL-producing *K. pneumoniae* (clinical strain) + 5 µl of f-hAM homogenate vs. ESBL-producing *K. pneumoniae*(reference strain) + 10 µl of c-hAM homogenate	> 0.9999
ESBL-producing *K. pneumoniae* (clinical strain) + 5 µl of f-hAM homogenate vs. ESBL-producing *K. pneumoniae*(clinical strain) + 5 µl of c-hAM homogenate	> 0.9999
ESBL-producing *K. pneumoniae* (clinical strain) + 5 µl of f-hAM homogenate vs. ESBL-producing *K. pneumoniae* (clinical strain) + 10 µl of c-hAM homogenate	> 0.9999
ESBL-producing *K. pneumoniae* (clinical strain) + 10 µl of f-hAM homogenate vs. A. *baumannii* (reference strain) + 5 µl of c-hAM homogenate	> 0.9999
ESBL-producing *K. pneumoniae* (clinical strain) + 10 µl of f-hAM homogenate vs. A. *baumannii*(reference strain) + 10 µl of c-hAM homogenate	0.2861
ESBL-producing *K. pneumoniae* (clinical strain) + 10 µl of f-hAM homogenate vs. A. *baumannii*(clinical strain) + 5 µl of c-hAM homogenate	> 0.9999
ESBL-producing *K. pneumoniae* (clinical strain) + 10 µl of f-hAM homogenate vs. *A. baumannii* (clinical strain) + 10 µl of c-hAM homogenate	> 0.9999
ESBL-producing *K. pneumoniae* (clinical strain) + 10 µl of f-hAM homogenate vs. ESBL-producing *E. coli* (clinical strain) + 5 µl of c-hAM homogenate	> 0.9999
ESBL-producing *K. pneumoniae* (clinical strain) + 10 µl of f-hAM homogenate vs. ESBL-producing *E. coli* (clinical strain) + 10 µl of c-hAM homogenate	> 0.9999
ESBL-producing *K. pneumoniae* (clinical strain) + 10 µl of f-hAM homogenate vs. ESBL-producing*K. pneumoniae*(reference strain) + 5 µl of c-hAM homogenate	> 0.9999
ESBL-producing *K. pneumoniae* (clinical strain) + 10 µl of f-hAM homogenate vs. ESBL-producing *K. pneumoniae*(reference strain) + 10 µl of c-hAM homogenate	> 0.9999
ESBL-producing *K. pneumoniae* (clinical strain) + 10 µl of f-hAM homogenate vs. ESBL-producing *K. pneumoniae*(clinical strain) + 5 µl of c-hAM homogenate	> 0.9999
ESBL-producing *K. pneumoniae* (clinical strain) + 10 µl of f-hAM homogenate vs. ESBL-producing *K. pneumoniae* (clinical strain) + 10 µl of c-hAM homogenate	> 0.9999
A. *baumannii* (reference strain) + 5 µl of c-hAM homogenate vs. A. *baumannii*(reference strain) + 10 µl of c-hAM homogenate	> 0.9999
A. *baumannii* (reference strain) + 5 µl of c-hAM homogenate vs. A. *baumannii*(clinical strain) + 5 µl of c-hAM homogenate	0.0575
A. *baumannii* (reference strain) + 5 µl of c-hAM homogenate vs. *A. baumannii* (clinical strain) + 10 µl of c-hAM homogenate	> 0.9999
A. *baumannii* (reference strain) + 5 µl of c-hAM homogenate vs. ESBL-producing *E. coli* (clinical strain) + 5 µl of c-hAM homogenate	> 0.9999
A. *baumannii* (reference strain) + 5 µl of c-hAM homogenate vs. ESBL-producing *E. coli* (clinical strain) + 10 µl of c-hAM homogenate	> 0.9999
A. *baumannii* (reference strain) + 5 µl of c-hAM homogenate vs. ESBL-producing*K. pneumoniae*(reference strain) + 5 µl of c-hAM homogenate	0.1929
A. *baumannii* (reference strain) + 5 µl of c-hAM homogenate vs. ESBL-producing *K. pneumoniae*(reference strain) + 10 µl of c-hAM homogenate	> 0.9999
A. *baumannii* (reference strain) + 5 µl of c-hAM homogenate vs. ESBL-producing *K. pneumoniae*(clinical strain) + 5 µl of c-hAM homogenate	0.0485
A. *baumannii* (reference strain) + 5 µl of c-hAM homogenate vs. ESBL-producing *K. pneumoniae* (clinical strain) + 10 µl of c-hAM homogenate	> 0.9999
A. *baumannii*(reference strain) + 10 µl of c-hAM homogenate vs. A. *baumannii*(clinical strain) + 5 µl of c-hAM homogenate	< 0.0001
A. *baumannii*(reference strain) + 10 µl of c-hAM homogenate vs. *A. baumannii* (clinical strain) + 10 µl of c-hAM homogenate	> 0.9999
A. *baumannii*(reference strain) + 10 µl of c-hAM homogenate vs. ESBL-producing *E. coli* (clinical strain) + 5 µl of c-hAM homogenate	0.0001
A. *baumannii*(reference strain) + 10 µl of c-hAM homogenate vs. ESBL-producing *E. coli* (clinical strain) + 10 µl of c-hAM homogenate	> 0.9999
A. *baumannii*(reference strain) + 10 µl of c-hAM homogenate vs. ESBL-producing*K. pneumoniae*(reference strain) + 5 µl of c-hAM homogenate	0.0004
A. *baumannii*(reference strain) + 10 µl of c-hAM homogenate vs. ESBL-producing *K. pneumoniae*(reference strain) + 10 µl of c-hAM homogenate	0.5348

A. *baumannii*(reference strain) + 10 µl of c-hAM homogenate vs. ESBL-producing *K. pneumoniae*(clinical strain) + 5 µl of c-hAM homogenate	< 0.0001
A. *baumannii*(reference strain) + 10 µl of c-hAM homogenate vs. ESBL-producing *K. pneumoniae* (clinical strain) + 10 µl of c-hAM homogenate	0.9246
A. *baumannii*(clinical strain) + 5 µl of c-hAM homogenate vs. *A. baumannii* (clinical strain) + 10 µl of c-hAM homogenate	0.6219
A. *baumannii*(clinical strain) + 5 µl of c-hAM homogenate vs. ESBL-producing *E. coli* (clinical strain) + 5 µl of c-hAM homogenate	> 0.9999
A. *baumannii*(clinical strain) + 5 µl of c-hAM homogenate vs. ESBL-producing *E. coli* (clinical strain) + 10 µl of c-hAM homogenate	0.0513
A. *baumannii*(clinical strain) + 5 µl of c-hAM homogenate vs. ESBL-producing*K. pneumoniae*(reference strain) + 5 µl of c-hAM homogenate	> 0.9999
A. *baumannii*(clinical strain) + 5 µl of c-hAM homogenate vs. ESBL-producing *K. pneumoniae*(reference strain) + 10 µl of c-hAM homogenate	> 0.9999
A. *baumannii*(clinical strain) + 5 µl of c-hAM homogenate vs. ESBL-producing *K. pneumoniae*(clinical strain) + 5 µl of c-hAM homogenate	> 0.9999
A. *baumannii*(clinical strain) + 5 µl of c-hAM homogenate vs. ESBL-producing *K. pneumoniae* (clinical strain) + 10 µl of c-hAM homogenate	> 0.9999
A. baumannii (clinical strain) + 10 µl of c-hAM homogenate vs. ESBL-producing *E. coli* (clinical strain) + 5 µl of c-hAM homogenate	> 0.9999
A. baumannii (clinical strain) + 10 µl of c-hAM homogenate vs. ESBL-producing *E. coli* (clinical strain) + 10 µl of c-hAM homogenate	> 0.9999
A. baumannii (clinical strain) + 10 µl of c-hAM homogenate vs. ESBL-producing*K. pneumoniae*(reference strain) + 5 µl of c-hAM homogenate	0.7758
A. baumannii (clinical strain) + 10 µl of c-hAM homogenate vs. ESBL-producing *K. pneumoniae*(reference strain) + 10 µl of c-hAM homogenate	> 0.9999
A. baumannii (clinical strain) + 10 µl of c-hAM homogenate vs. ESBL-producing *K. pneumoniae*(clinical strain) + 5 µl of c-hAM homogenate	0.2348
A. baumannii (clinical strain) + 10 µl of c-hAM homogenate vs. ESBL-producing *K. pneumoniae* (clinical strain) + 10 µl of c-hAM homogenate	> 0.9999
ESBL-producing *E. coli* (clinical strain) + 5 µl of c-hAM homogenate vs. ESBL-producing *E. coli* (clinical strain) + 10 µl of c-hAM homogenate	> 0.9999
ESBL-producing *E. coli* (clinical strain) + 5 µl of c-hAM homogenate vs. ESBL-producing*K. pneumoniae*(reference strain) + 5 µl of c-hAM homogenate	> 0.9999
ESBL-producing *E. coli* (clinical strain) + 5 µl of c-hAM homogenate vs. ESBL-producing *K. pneumoniae*(reference strain) + 10 µl of c-hAM homogenate	> 0.9999
ESBL-producing *E. coli* (clinical strain) + 5 µl of c-hAM homogenate vs. ESBL-producing *K. pneumoniae*(clinical strain) + 5 µl of c-hAM homogenate	> 0.9999
ESBL-producing *E. coli* (clinical strain) + 5 µl of c-hAM homogenate vs. ESBL-producing *K. pneumoniae* (clinical strain) + 10 µl of c-hAM homogenate	> 0.9999
ESBL-producing *E. coli* (clinical strain) + 10 µl of c-hAM homogenate vs. ESBL-producing*K. pneumoniae*(reference strain) + 5 µl of c-hAM homogenate	0.1795
ESBL-producing *E. coli* (clinical strain) + 10 µl of c-hAM homogenate vs. ESBL-producing *K. pneumoniae*(reference strain) + 10 µl of c-hAM homogenate	> 0.9999
ESBL-producing *E. coli* (clinical strain) + 10 µl of c-hAM homogenate vs. ESBL-producing *K. pneumoniae*(clinical strain) + 5 µl of c-hAM homogenate	0.0449
ESBL-producing *E. coli* (clinical strain) + 10 µl of c-hAM homogenate vs. ESBL-producing *K. pneumoniae* (clinical strain) + 10 µl of c-hAM homogenate	> 0.9999
ESBL-producing*K. pneumoniae*(reference strain) + 5 µl of c-hAM homogenate vs. ESBL-producing *K. pneumoniae*(reference strain) + 10 µl of c-hAM homogenate	> 0.9999
ESBL-producing*K. pneumoniae*(reference strain) + 5 µl of c-hAM homogenate vs. ESBL-producing *K. pneumoniae*(clinical strain) + 5 µl of c-hAM homogenate	> 0.9999
ESBL-producing*K. pneumoniae*(reference strain) + 5 µl of c-hAM homogenate vs. ESBL-producing *K. pneumoniae* (clinical strain) + 10 µl of c-hAM homogenate	> 0.9999
ESBL-producing *K. pneumoniae*(reference strain) + 10 µl of c-hAM homogenate vs. ESBL-producing *K. pneumoniae*(clinical strain) + 5 µl of c-hAM homogenate	> 0.9999

ESBL-producing *K. pneumoniae*(reference strain) + 10 μl of c-hAM homogenate vs. ESBL-producing *K. pneumoniae* (clinical strain) + 10 μl of c-hAM homogenate	> 0.9999
ESBL-producing *K. pneumoniae*(clinical strain) + 5 μl of c-hAM homogenate vs. ESBL-producing *K. pneumoniae* (clinical strain) + 10 μl of c-hAM homogenate	> 0.9999
